# What is the value of testing for tick-borne diseases in cattle in endemic areas? A case study of bovine anaplasmosis

**DOI:** 10.1371/journal.pone.0315202

**Published:** 2025-03-12

**Authors:** Valeria Paucar-Quishpe, Dirk Berkvens, Ximena Pérez-Otáñez, Richar Rodríguez-Hidalgo, Darío Cepeda-Bastidas, Cecilia Perez, Yadira Guasumba, Daniela Balseca, Kamilo Villareal, María-Augusta Chávez-Larrea, Sandra Enríquez, Jorge Grijalva, Sophie O. Vanwambeke, Claude Saegerman, Lenin Ron-Garrido

**Affiliations:** 1 Research Unit of Epidemiology and Risk Analysis applied to Veterinary Science (UREAR-ULiège), Fundamental and Applied Research for Animals & Health (FARAH) Center/Faculty of Veterinary Medicine, University of Liege, Liège, Belgium; 2 Instituto de Investigación en Zoonosis (CIZ), Universidad Central del Ecuador, Quito, Ecuador; 3 Department of Biomedical Sciences, Institute of Tropical Medicine, Antwerp, Belgium; 4 Georges Lemaitre Centre for Earth and Climate Research, Earth & Life Institute, UCLouvain, Louvain-la-Neuve, Belgium; 5 Facultad de Medicina Veterinaria y Zootecnia, Universidad Central del Ecuador, Quito, Ecuador; 6 Facultad de Ciencias Agrícolas, Universidad Central del Ecuador, Quito, Ecuador; 7 Centro de Biología, Universidad Central del Ecuador. Quito, Ecuador; 8 Facultad de Ciencias Químicas, Universidad Central del Ecuador. Quito, Ecuador; 9 Departamento de Ciencias de la Vida y la Agricultura, Grupo de Investigación en Sanidad Animal y Humana (GISAH), Carrera de Ingeniería en Biotecnología, Universidad de las Fuerzas Armadas ESPE, Sangolquí, Ecuador; National Veterinary Research Institute (NVRI), NIGERIA

## Abstract

Anaplasmosis is a tick-borne disease (TBDs) caused by *Anaplasma* spp. In areas where TBDs are endemic, it is crucial to consider the animals’ immunological status in relation to these diseases. The true prevalence of bovine anaplasmosis, the percentage of animals with protective antibodies against this TBD, and the diagnostic characteristics of three tests (multiplex polymerase chain reaction (mPCR), competitive-inhibition enzyme-linked immunosorbent assay (cELISA), and blood smear (BS)) were estimated using a Bayesian approach. A total of 620 samples were collected in two subtropical areas of Ecuador. A significant finding of this study is that approximately 70% of cattle in those endemic areas harbored protective antibodies against *Anaplasma marginale*. This elevated percentage may stem from persistent exposure with a high pathogen prevalence in ticks. The decline in cELISA specificity must be attributed to cross-reactivity with protective antibodies against *Anaplasma* spp. It is crucial to interpret this test outcome alongside exposure history and clinical manifestations. The elevated apparent prevalence detected by cELISA and BS should be contextualized with mPCR results. The high seroprevalence and infrequent clinical outbreaks suggest that the pathogen has achieved endemic stability. This study provides valuable insights into the dynamics of anaplasmosis in endemic areas and may serve as a foundation for devising TBDs control strategies in these areas.

## Introduction

Diseases transmitted by ticks in cattle, called tick-borne diseases (TBDs), occur throughout the world, especially in tropical and subtropical areas [1]. Bovine anaplasmosis is caused by obligate intra-erythrocytic bacteria called *Anaplasma*. The main species responsible of the disease in cattle are *Anaplasma marginale* and, to a lesser degree, *Anaplasma centrale* [2]. This disease is usually transmitted by tick bites (*Rhipicephalus microplus*). Haematophagous insects, such as horse flies (*Tabanus* spp.), mosquitoes (*Psorophora* spp.), stable fly (*Stomoxys calcitrans*), *Haematobia irritans* and fomites (surgical instruments, or needles contaminated with blood) are also responsible for the transmission [3–5]. In areas of the USA, Africa, Central and South, where there are no tick vectors, mechanical transmission by *Tabanus* (horseflies) specimens is the main route of the spread of *A. marginale* [6,7]. Vertical transmission has also been mentioned to occur under conditions of constant inoculation in endemic areas [8,9].

Epidemiologically, three types of zones are recognised in relation to the local disease status of tick-borne diseases: free, stable, and unstable zones. Free zones are those in which conditions are not favourable for vector development. Epidemiologically unstable zones are areas where the occurrence of the disease is determined by climatic conditions and/or livestock management. Stable zones are zones where animals have sufficient antibody levels to guarantee their protection and an asymptomatic disease is present [10–14]. Although clinical signs and necropsy findings can guide the diagnosis of anaplasmosis, techniques that identify the presence of the pathogen are indispensable, particularly in unstable and epidemiologically free zones. Serious losses used occur when mature cattle with no previous exposure are moved into endemic areas or when animals are in endemically unstable areas [15].

All naïve animals are susceptible to the disease; however, clinical manifestations increase according to host factors: low immune status, cattle breed (*Bos taurus*), and host age (adult animals). Bovine anaplasmosis is characterized by fever, depression, weakness, icterus, anaemia, and often death in adult animals [5,16–18]. Calves rarely develop clinical signs of acute disease in endemic areas. Following recovery from the clinical phase, animals may remain asymptomatic, persistently carrying the infection (challenge-immunized animals) [15,19–21].

Infections can be detected directly by PCR or blood smears or indirectly by serology [22]. The most common and routine technique for diagnosis is the microscopic examination of blood or organ smears (liver, kidney, heart, or lungs) with Giemsa stain. However, it only detects animals with acute infections given that chronic-phase infections do not express a high level of parasitaemia [23,24]. On the other hand, although PCR allows us to determine the agent’s presence and differentiate the species of *Anaplasma* present, it is a technique that cannot be performed on a large scale, achieved with serological tests [25]; high costs can make this test impractical, particularly when large number of samples are collected to estimate infection prevalence at animal level. A potential solution involves screening a large number of farms through pool testing, wherein sample pools are screened, making use of the high sensitivity and specificity of PCR; and when a pool is detected as positive, the positive individual sample(s) is (are) then sought within the original constituent samples [26]. Pooled testing provides a cost-effective alternative to testing individual animal samples. The accuracy of estimates obtained through pooled testing relies on factors such as the true prevalence, pool size, and number of pooled tests used [27–29].

Serological assays for the anaplasmosis diagnosis include card agglutination test (CAT), indirect fluorescence antibody test (IFAT), and complement fixation tests (CFT), enzyme-linked immunosorbent assay (ELISA) including a competitive ELISA (cELISA) and indirect ELISA (iELISA) [2]. Competitive ELISA and indirect ELISA use antigens with strong specificity and high sensitivity to detect IgG and IgM antibodies which may vary depending on several factors, such as exposure time, the pathogens or strains of *Anaplasma* spp. involved and the infection status of the animals [30]. The two serological tests currently preferred for diagnosing infected animals are cELISA and CAT [2,23].

Imperfect sensitivity and specificity of existing diagnostic tests can lead to misclassification of some animals tested [31,32]. Therefore, to estimate test characteristics under field conditions, sensitivity and specificity values should be obtained from prior information data or in case of absence, from expert opinion (*prior distribution*). Bayesian statistics allows us to determine the likelihood function using prior information about the parameters available in the observed data. Combining both the a priori distribution and the likelihood function using Bayes’ theorem yields a *posterior distribution*, which reflects updated knowledge [33,34]. In the context of epidemiology and diagnostic tests, Berkvens et al. (2006) [35] described a Bayesian approach using probabilistic constraints to estimate true disease prevalence and diagnostic test characteristics when a set of diagnostic tests is applied to a set of individuals in a population. However, the issue of employing pooled testing in conjunction with individual assays has yet to be fully evaluated.

Concerning TBDs and the characteristics of the tests used for their detection in epidemiologically stable areas for anaplasmosis, what do the diagnostic test results mean for the conclusions about the health status of the animals? In theory, an animal is considered to have anaplasmosis when it exhibits clinical signs consistent with the disease caused by the pathogen for which it tested positive. In tests that detect antibodies, seropositivity alone does not necessarily indicate current illness; it signifies exposure to the pathogen and the development of an immune response. However, if an animal is either seropositive or negative, and exhibits clinical signs that correspond to the disease, it suggests an active infection or anaplasmosis [2]. For this reason, it is important to interpret the test results carefully and correlate them with the animal’s clinical signs and history of exposure to make an accurate diagnosis and determine the appropriate course of treatment or management [5,36,37]. In endemic areas, cattle usually become infected with *A. marginale* in early life, and losses due to anaplasmosis are minimal; similarly, these cattle remain as chronically infected carriers, allowing them to develop lifelong active immunity (challenge-immunized) without clinical disease [10,15]. Additionally, cows have high enough levels of antibodies to promote protection that can be passed to calves with the colostrum [12]. Thus, the objective of this study was to evaluate the diagnostic performance (sensitivity and specificity) of three diagnostic tests (cELISA, PCR, and BS) used for the detection of anaplasmosis in endemic dairy farms in Ecuador, applying a Bayesian modeling approach. To fulfill this objective, we built a conditional probabilistic model that allows the incorporation of a two-step approach for PCR. In this process, the initial PCR step is applied to pooled samples per farm, followed by a secondary step conducted only when a positive result emerges from the pooled sample, at which point individual animal testing ensues.

## Materials and methods

### Ethics statement

This study was approved by the Research Committee of the Faculty of Veterinary Medicine and Zootechnics (COIF-FMVZ) of the *Universidad Central del Ecuador* (UCE-FMVZ-DEC-2023-0631-O). All animals were treated with care, and the usual farm management for blood sample collection was followed. As the procedure was a routine blood extraction, anaesthesia or analgesia was not required. Veterinarians conducted the sample collection, animals were not mistreated, and animal welfare was guaranteed. Adequate information was provided to farmers, who provided written consent before the commencement of sample collection from their animals. Each farm surveyed was assigned a unique code to ensure anonymity, incorporating numbers and letters to denote the specific farm and area visited.

### Study area and sampling design

**Fig 1:**
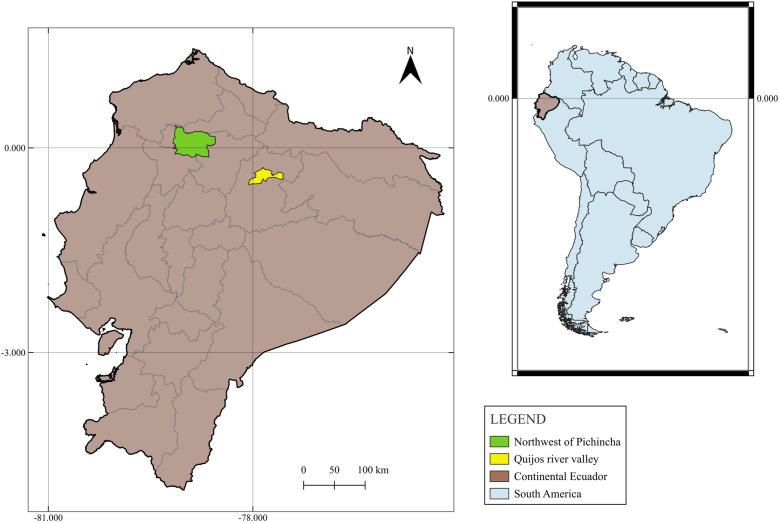
Study areas. Legend: Images were created by the author using data from [45,46]. Published under the CC BY 4.0 license.

This study was part of the project “Socio-eco-epidemiology of ticks, tick-borne parasites, acaricide resistance and residual effects of acaricides in Ecuadorian tropical livestock: environmental, animal and public health impacts”. Between November 2020 and March 2021, a cross-sectional survey was conducted in two sub-tropical areas of Ecuador (**Fig 1**).

Area 1: Northwest of Pichincha Province, situated in the western foothills of the Andes (Coastal Region) between 0°18N, 79°10W and 0°09S, 78°33W. This zone experiences temperatures ranging from 19°C to 25°C at 600 metres above sea level (m.a.s.l.) to 1800 m.a.s.l [38,39] and is crossed by Chocó Andino of Pichincha Biosphere Reserve [40]. Area 2: Quijos River Valley, located in the eastern foothills of the Andes (Amazon region) between 0°18S, 78°04W and 0°33S, 77°32W. Here, temperatures fluctuate between 16 and 24°C, with an altitude ranging from 1500 to 2000 m.a.s.l [41–43]. This area is in the middle of two protected areas, Cayambe Coca National Park and Sumaco Napo Galeras National Park [44]. Five randomly selected animals were sampled at each farm without distinction of breed, age or sex. A total of 695 animals from 139 farms were sampled. Parameters such as temperature (°C), capillary refill time, heart rate, and respiratory rate were also recorded during clinical examination.

### Processing of blood samples

Two tubes with about 4 ml of blood were collected from each animal by coccygeal venipuncture with disposable needles and plastic Vacutainer^TM^ tubes (red top, no additives; and purple top, EDTA as an anticoagulant). Each blood sample was assigned a unique code, incorporating numbers and letters to denote the specific farm and area visited (animal code). Blood sample processing was done at the immunodiagnostic laboratory at the *Instituto de Investigación en Zoonosis* (CIZ) at the *Universidad Central del Ecuador*. Sera collected in red top Vacutainer^TM^ tubes were separated by centrifugation (250 rpm). Each serum was stored in respectively labeled graduated cryovials. Haemolysed, lipemic, icteric, and contaminated samples were removed from the study [47]. Animals with only one or two test results and/or farms where less than five animals were sampled are also excluded from the study. A cured and consolidated database was obtained with the results of 620 animals (89% sampled) belonging to 124 farms (89% sampled).

### Microscopic examination

**Fig 2:**
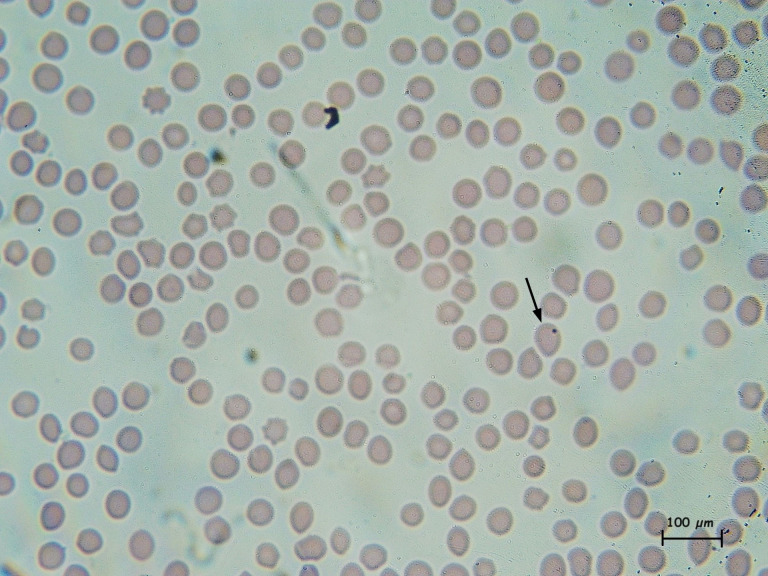
Giemsa-stained blood smear showing *Anaplasma* spp. in bovine erythrocytes of cattle. Legend: Photo credit, Kamilo Villarreal, Clinical Biochemistry Department, Faculty of Chemical Sciences, Universidad Central del Ecuador.

The microscopic examination consisted of the identification of the bacterium (*Anaplasma* spp.) in erythrocytes on blood smears (BS) stained with Giemsa. Blood samples collected in EDTA Vacutainer^TM^ tubes were used to prepare thin BS. For each sample, two BS slides were made and identified with the animal code. The BS were fixed in methanol for 3 min and stained in Giemsa stain diluted at 10% with buffer solution for 25 min. Each BS prepared was examined under × 100 objective magnification and oil immersion on a Better Scientific microscope. Thorough searches were performed for small, rounded, deep purple intra-erythrocytic inclusions, approximately 0.3-1.0 µm in diameter. More than 30 microscopic fields per slide were observed before considering an animal as negative; a positive animal had at least one positive BS (parallel testing) [23,48–51]. A total of 1240 BS from 620 animals were examined for intra-erythrocytic inclusions using Giemsa stain. **Fig 2** presents the result of a blood smear positive test.

### Serological tests

A competitive enzyme-linked immunosorbent assay (cELISA) based on antibody binding to major surface protein 5 (MSP5) of *A. marginale* was used. Sera were tested for specific antibodies against *A. marginale* using a commercial ELISA kit, *Anaplasma* Antibody Test Kit cELISA v2 (Veterinary Medical Research and Development; VMRD, Inc.), following the manufacturer’s instructions, catalog number 283–2 (S1 Text). Receiver operator characteristics (ROC) analysis was performed to determine the optimal cut-off value. Bovine blood samples from a disease-free zone were used as negative controls (N = 70), and bovine blood samples positive for *A. marginale* by multiplex PCR (mPCR) were used as positive controls (N = 43). These samples were obtained from the Animal Serum Bank of CIZ, Centro Experimental Uyumbicho of the Universidad Central del Ecuador, and the “*Hacienda El Prado Selva Alegre*” of the *Universidad de las Fuerzas Armadas* (ESPE). The analysis was performed using R [52], package cutpointr [53]. The optimal cut-off was 35.76 with high sensitivity and specificity, 99 to 100%, respectively.

### Molecular

Bovine blood samples were organised into pools of 5 individuals (*k* =  5) per farm. The testing procedure was a two-step approach: the first step consisted of applying a PCR to a pooled sample per farm. If the result of this test was negative, all the animals were declared negative. Conversely, if the result of the pooled test was positive, then the blood sample of each animal in the pool was individually tested by PCR.

### DNA extraction

Genomic DNA was extracted from 400 µ L of blood pools using the Column Pure Blood Genomic DNA kit (Applied Biological Materials Inc., Canada) in accordance with the manufacturer’s instructions. The DNA was eluted in 60 µ L of elution buffer and stored at -20°C until use.

### Multiplex PCR for amplification of Anaplasma marginale and Anaplasma centrale

Based on previous studies, a set of specific primers for *Anaplasma* spp. were selected (**Table 1**). For the detection of *Anaplasma marginale* and *Anaplasma centrale*, the msp5 (714 bp) and msp4 (395 bp) genes were targeted, respectively [54,55].

**Table 1:** List of PCR primers used in the present study.

Parasite	Gene target	Name	Primer sequence 5’ – 3’	Product size (bp)	Reference
*A. marginale*	*msp5*	Ana 19A	GTGTTCCTGGGGTACTCCTA	714	[54]
*A. marginale*	*msp5*	Ana 19B	TGATCTGGTCAGCCCCAGCT	714	
*A. centrale*	*msp4*	CentF	CATGGGGCATGAATCTGTG	395	[55]
*A. centrale*	*msp4*	CentR	AATTGGTTGCAGTGAGCGC	395	

The multiplex PCR reaction was performed as described by Guasumba (2022) [56] (S2 Text).

### Individual PCR of Anaplasma marginale

Positive pools for *Anaplasma marginale* were subjected to individual PCR as described by Guasumba (2022) [56] (S2 Text).

### Conditional-probabilistic model description

The Bayesian analysis approach described by Berkvens et al. (2006) [35] and Branscum et al. (2005) [57] was used in WinBUGS 1.4.3 [58] to estimate the sensitivities and specificities of the three tests and the true prevalence of anaplasmosis in cattle from subtropical areas of Ecuador. Additional calculations were performed in R version 4.2.0 [52].

For our analysis, results of anaplasmosis diagnosis tests were sorted in the following order: mPCR (T1), cELISA (T2), and BS (T3). Tests were considered conditionally dependent. Under this assumption, a multinomial model including all possible interactions between the three tests requires 15 parameters (Ɵ_1_-Ɵ_15_) to be estimated. These included the prevalence (Ɵ_1_), sensitivity (Ɵ_2_), and specificity (Ɵ_3_) of the T1; two conditional sensitivities and two conditional specificities of the T2; and four conditional sensitivities and four conditional specificities for the T3 (S3 Text).

Due to the two-step approach practiced for the pool testing (PCR), each test sample that is part of a pool can either be truly positive or truly negative [29]. Using the methodology described in detail by Speybroeck et al., 2012 [29] and given a fixed pool sample size (represented by *k*), the calculation of the apparent prevalence (Ɵ_1_’) will depend on the probability of at least one positive result in the pool as showed in Equation 1:


θ1'=θ11−1−θ1k (1)

Sensitivity of PCR pool testing (Ɵ2’) is now dependent of the number of positives in the group and from the PCR-test sensitivity (Ɵ_2_) as shown in Equation 2:


θ2'=∑i=1k1−1−θ2ik!i!k−i!θ1i1−θ1k−i1−1−θ1k (2)

Thus, a combination of the two previous results represents the true positive rate under pool testing is:


ϑ=θ1'θ2' (3)

Considering the outcome of the three tests (**Table 2**), we have:

**Table 2:** The contingency table of the three tests.

	cELISA +		cELISA-	
	BS +	BS ^-^	BS +	BS ^-^
mPCR +	T1 + T2 + T3 + 111	T1 + T2 + T3^-^ 110	T1 + T2^-^ T3 + 101	T1 + T2^-^ T3^-^ 100
mPCR^-^	T1^-^ T2 + T3 + 011	T1^-^ T2 + T3^-^ 010	T1^-^ T2^-^ T3 + 001	T1^-^ T2^-^ T3^-^ 000

Multiplex PCR, mPCR (T1); Competitive-inhibition enzyme-linked immunosorbent assay, cELISA (T2); Blood smear, BS (T3). Being negative results (0 or -), and positive results (1 or + ), the expected cell probabilities under the conditional dependence.

Denoting by 0 negative results, positive test outcomes by 1, the expected cell probabilities (*P*) based on these three tests under the conditional dependence (the different conditional probabilities are listed in S3 Text) assumption are:


$$P\left(111\right)=\vartheta \theta_{4}\theta_{8}+\left(1-\vartheta \right)(1-\theta_{3})(1-\theta_{7})\left(1-\theta_{15}\right)$$ (4)

$$P\left(110\right)=\vartheta \theta_{2}\theta_{4}(1-\theta_{8})+\left(1-\vartheta \right)(1-\theta_{3})(1-\theta_{7})\theta_{15}$$ (5)

$$P\left(101\right)=\vartheta \theta_{2}\left(1-\theta_{4}\right)\theta_{9}+\left(1-\vartheta \right)(1-\theta_{3})\theta_{7}\left(1-\theta_{14}\right)$$ (6)

$$P\left(100\right)=\vartheta \theta_{2}\left(1-\theta_{4}\right)(1-\theta_{9})+\left(1-\vartheta \right)(1-\theta_{3})\theta_{7}\theta_{14}$$ (7)

$$P\left(011\right)=\vartheta (1-\theta_{2})\theta_{5}\theta_{10}+\left(1-\vartheta \right)\theta_{3}(1-\theta_{6})\left(1-\theta_{13}\right)$$ (8)

$$P\left(010\right)=\vartheta (1-\theta_{2})\theta_{5}(1-\theta_{10})+\left(1-\vartheta \right)\theta_{3}(1-\theta_{6})\theta_{13}$$ (9)

$$P\left(001\right)=\vartheta (1-\theta_{2})\left(1-\theta_{5}\right)\theta_{11}+\left(1-\vartheta \right)\theta_{3}\theta_{6}(1-\theta_{12})$$ (10)

$$P\left(000\right)=\vartheta (1-\theta_{2})\left(1-\theta_{5}\right)(1-\theta_{11})+\left(1-\vartheta \right)\theta_{3}\theta_{6}\theta_{12}$$ (11)

In addition, animals that were tested positive for cELISA results but remained asymptomatic were termed challenged-immunized animals, with their proportion calculated (S4 Text) as follows:


$$\frac{numberofcELISAfalsepositives}{numberofcELISAfalsepositives+numberofcELISAtruepositives}$$ (12)

Different Bayesian models with different a priori information and assumptions for the estimation of the parameters of the multinomial model were used in conjunction with their evaluation criteria under Bayesian statistics:

• **M1**: specificity of T1 = 1
• **M2**: specificity of T1 = 1, the sensitivity of T1 constrained uniformly (dunif[0.95,1.00]), beta prior on Ɵ_12_ in 0.98 (dbeta[235,5])
• **M3**: specificity of T1 = 1, Ɵ_6_ = 1
• **M4**: specificity of T1 = 1, Ɵ_6_ = 1, beta prior on Ɵ_12_ in 0.98 (dbeta[235,5])
• **M5**: specificity of T1 = 1, sensitivity of T1 constrained uniformly (dunif[0.95,1.00]), Ɵ_12_ = 1
• **M6**: specificity of T1 constrained uniformly (dunif[0.99,1.00]), the sensitivity of T1 constrained uniformly (dunif[0.95,1.00]), beta prior on Ɵ_12_ in 0.98 (dbeta[235, 5])
• **M7**: specificity of T1 = 1, Ɵ_6_ =  1, uniform prior on Ɵ_5_ =  dunif[0.6, 1.00], beta prior on Ɵ_12_ in 0.98 (dbeta[235, 5])
• **M8**: sensitivity of T1 constrained uniformly (dunif[0.95, 1.00]), uniform prior on Ɵ_4_ = dunif [0.8, 1.00], uniform prior on Ɵ_5_ = dunif[0.8,1.00], Ɵ_6_ =  1, beta prior on Ɵ_12_ in 0.98 (dbeta[235, 5])

A summary of the models designed for this study with elicitation of the prior distributions is shown in **Table 3**.

**Table 3:** Models (M1 to M8) constructed and prior distributions used.

	M1	M2	M3	M4	M5	M6	M7	M8
theta [1]	0, 1	0, 1	0, 1	0, 1	0, 1	0, 1	0, 1	0, 1
theta [2]	0, 1	0.95, 1	0, 1	0, 1	0.95, 1	0.95, 1	0, 1	0.95, 1
theta [3]	1	1	1	1	1	0.99, 1	1	0, 1
theta [4]	0, 1	0, 1	0, 1	0, 1	0, 1	0, 1	0, 1	0.8, 1
theta [5]	0, 1	0, 1	0, 1	0, 1	0, 1	0, 1	0.6, 1	0.8, 1
theta [6]	0, 1	0, 1	1	1	0, 1	0, 1	1	1
theta [7]	–	–	–	–	–	0, 1	–	0, 1
theta [8]	0, 1	0, 1	0, 1	0, 1	0, 1	0, 1	0, 1	0, 1
theta [9]	0, 1	0, 1	0, 1	0, 1	0, 1	0, 1	0, 1	0, 1
theta [10]	0, 1	0, 1	0, 1	0, 1	0, 1	0, 1	0, 1	0, 1
theta [11]	0, 1	0, 1	0, 1	0, 1	0, 1	0, 1	0, 1	0, 1
theta [12]	0, 1	235, 5^a^	0, 1	235, 5^a^	1	235, 5^a^	235, 5^a^	235, 5^a^
theta [13]	0, 1	0, 1	–	–	0, 1	0, 1	–	–
theta [14]	–	–	–	–	–	0, 1	–	0, 1
theta [15]	–	–	–	–	–	0, 1	–	0, 1

Prior distributions are uniform (dunif), excluded for ^a^ than are beta distribution (dbeta)

### Prior information for parameters

Estimations of the prevalence and test characteristics (sensitivities and specificities) of the three tests used are limited. Prior information was therefore introduced based on similar studies of bovine anaplasmosis in different countries found in the literature. The sensitivity and specificity of PCR were 95.2% (95% CI: 85.2–99.1%) and 92.7% (95% CI:85.6–99.2%), respectively [16]. For some parameters, no available or objective prior information can be formulated, and it is necessary to leave prior information on these parameters as non-informative. In addition to these values, a priori information was also obtained from the consultation of expert opinion from the Department of Biomedical Sciences from the Institute of Tropical Medicine, Belgium; the Research unit of Epidemiology and Risk Analysis Applied to Veterinary Science from the University of Liege, Belgium; and from the Department of Biostatistics and Epidemiology at CIZ, Ecuador.

### Sensitivity analysis

The influence of the prior information on the posterior estimates was assessed using sensitivity analysis [57].

A scenario was used for the chosen priors: perturbations of the prior interval (estimate - 0.0001, estimate +  0.0001). This model was run with the same number of chains, and similar diagnostic criteria were performed.

### Model diagnostics

All models were run with a burn-in period of 10 000 iterations, and estimates were based on a further 40 000 iterations using three Markov chains. Model selection proceeded according to the methodology described by Berkvens et al. (2006) [35] using the effective number of estimated parameters (*pD*), Bayesian P value (P value), and Deviance Information Criterion (DIC) as validation criteria. Moreover, the convergence of the model was also evaluated by trace plots, Kernel density plots, Brooks-Gelman-Rubin (BGR) convergence statistic plots, and autocorrelation plots [35,59,60]. The WinBUGS codes used are presented in S5 Text.

### Assessment of agreement between the tests

The level of agreement between the three diagnostic tests was calculated by Cohen’s kappa coefficient for the agreement between each pair of tests. Using a ‘two-by-two’ contingency table. The kappa coefficient was calculated by using the psych package [61] under R environment. The following ranges were used for the interpretation of the kappa coefficient: poor agreement (<0.00), slight agreement (0.00–0.20), fair agreement (0.21–0.40), moderate agreement (0.41–0.60), substantial agreement (0.61–0.80), and almost perfect (0.81–1.00) [62]. In addition, the level of agreement was expressed in terms of indices of positive (P_pos_) and negative (P_neg_) agreement [63,64], and their Confidence intervals were calculated as described by Uebersax, 2018 [65].

## Results

### Descriptive results and agreement between the tests

The mPCR results showed that 226 (AP =  36%) of 620 samples were positive for *A. marginale*, with no evidence of *A. centrale*. Of the 620 samples tested by cELISA and BS, 610 (AP = 98%) and 500 (AP = 81%) were positive for *Anaplasma* spp., respectively. The cross-classified test results obtained from the three individual tests during the epidemiological study are presented in **Table 4**.

**Table 4:** Cross-classified test results for *Anaplasma* spp. in cattle of Ecuador based on mPCR, cELISA, and blood smear.

mPCR	cELISA	BS	Number of samples
1	1	1	185
1	1	0	41
1	0	1	0
1	0	0	0
0	1	1	315
0	1	0	69
0	0	1	0
0	0	0	10
		Total	**620**

mPCR, multiplex polymerase chain reaction; cELISA, competitive-inhibition enzyme-linked immunosorbent assay; BS, blood smear.

During the clinical examination, the average temperature of the sampled animals was 38.3°C. The average heart rate of the animals was 75 beats per minute (bpm), and their respiratory rate was recorded at 28 breaths per minute. A capillary refill time of 1 to 2 seconds (s) was observed in 85% of the animals.

The agreement and kappa value between cELISA–blood smear, cELISA–mPCR and mPCR – BS are shown in **Table 5**. The agreement level between cELISA with mPCR and BS tests were slight, in the other hand it was poor between the mPCR and BS. Concordance between cELISA - BS results was high (90%) in the positive results (P_pos_), whereas the agreement on negative test results (P_neg_) was low (15%). Concordances between cELISA - mPCR and mPCR- BS results were around 50% of the Ppos, whereas the agreement on Pneg was estimated to be 5% and 31%, respectively.

**Table 5:** Agreement between the tests.

Test	Kappa coefficient (95% CI)	P_pos_ (95% CI)	P_neg_ (95% CI)
cELISA-BS	0.13 (0.06-0.20)^a^	0.90 (0.88-0.92)	0.15 (0.07-0.24)
cELISA-mPCR	0.02 (0.01-0.03) ^a^	0.54 (0.50-0.58)	0.05 (0.02-0.08)
mPCR- BS	0.02 (-0.04-0.07) ^a^	0.51 (0.47-0.55)	0.31 (0.26-0.36)

mPCR, multiplex polymerase chain reaction; cELISA, competitive-inhibition enzyme-linked immunosorbent assay; BS, blood smear; CI, confidence interval; P_pos_, positive agreement; P_neg_, negative agreement; a, slight agreement.

### Model selection and posterior estimates

The summary of the model selection criteria and the Bayesian estimates of model parameters from applying models M1 to M8 are presented in **Table 6**. In the M1 model, the negative *pD* value suggests that the constraints are insufficient to estimate the model parameters. In models M3, M4, M7, and M8, the *pD* values calculated from the constraints imposed are positives and close to 3, which indicates that all parameters can be estimated. However, the Bayesian *P* values (1.00) suggest a lack-of-fit, which is also reflected in the badly estimated prevalence and sensitivities. In contrast, models M2, M5, and M6 have correct *pD* values, and the prevalences almost equal their true values. Model M5 has a Bayesian *P* value close to 0.5, the distribution kernel density (stemmed unimodal) and autocorrelation graphs showed good convergence (S6 Text), and it has the lowest DIC value of the three models, in addition, the DIC of this model was lower than 36.81, the maximum/optimum value obtainable from the data.

**Table 6:** Comparison of model diagnostic parameters used to estimate the true prevalence of anaplasmosis in cattle and sensitivity and specificity of three diagnostic tests.

Model	*P* value	DIC	*pD*	Prevalence	mPRC		cELISA		BS	
**Se**	**Sp**	**Se**	**Sp**	**Se**	**Sp**
M1	0.68	21.10	-10.03	0.47	0.81	1.00	0.98	0.03	0.79	0.21
M2	0.58	32.83	3.42	0.32	0.98	1.00	0.99	0.03	0.81	0.20
M3	1.00	72.23	3.07	1.00	0.45	1.00	0.99	1.00	0.82	0.91
M4	1.00	70.69	2.98	1.00	0.45	1.00	1.00	1.00	0.82	0.98
M5	0.56	32.58	3.40	0.32	0.98	1.00	0.99	0.02	0.80	0.20
M6	0.61	33.63	3.23	0.31	0.98	1.00	0.99	0.03	0.81	0.20
M7	1.00	70.68	2.97	1.00	0.45	1.00	1.00	1.00	0.82	0.98
M8	1.00	1556.02	3.00	0.98	0.95	0.90	1.00	0.94	0.81	0.93

mPCR, multiplex polymerase chain reaction; cELISA, competitive-inhibition enzyme-linked immunosorbent assay; BS, blood smear; M1 to M8, Bayesian models tested; Se, sensitivity; Sp, specificity; P value, Bayesian P value; DIC, Deviance Information Criterion; pD, the effective number of estimated parameters.

**Table 7** summarises the estimated values for Se and Sp for the three tests. Model 5 estimated a true prevalence of *Anaplasma marginale* to be 32% (95% CrI: 27% - 38%) and the proportion of challenge-immunized animals (PCIA) to be 67% (95% CrI: 62%-73%). The results of the sensitivity analyses are shown in **Table 8** (Model 5’), which introduces perturbations of ±  0.0001 to the limits of all prior intervals, shows only minimal changes in estimated parameter values and their associated 95% credible intervals. In addition to these statistically insignificant changes, the Bayes P and *pD* values tended to zero, which underscores the robustness of the model to such perturbations.

**Table 7:** Posterior mean for the prevalence and the test characteristics (Model 5).

Test	Parameter	Posterior estimates (95% CrI)
	Prevalence	0.32 (0.27-0.38)
**mPRC**	Se	0.98 (0.95-1.00)
	Sp	1.00
**cELISA**	Se	0.99 (0.97-1.00)
	Sp	0.02 (0.01-0.04)
**BS**	Se	0.80 (0.75-0.85)
	Sp	0.20 (0.16-0.24)

mPCR, multiplex polymerase chain reaction; cELISA, competitive-inhibition enzyme-linked immunosorbent assay; BS, blood smear; CrI, credibility interval; Se, sensitivity; Sp, specificity.

**Table 8:** Posterior mean for the prevalence and the test characteristics based on a sensitivity analysis.

Model & tests	*P* value	DIC	*pD*	Prevalence (95% CrI)	Sensitivity (95% CrI)	Specificity (95% CrI)
Model 5’	0.00	24.64	0.00	0.32 (0.32,0.32)		
mPRC					0.98 (0.98-0.98)	1
cELISA					0.99 (0.99-0.99)	0.02 (0.02-0.02)
BS					0.81 (0.80-0.81)	0.20 (0.20-0.20)

mPCR, multiplex polymerase chain reaction; cELISA, competitive-inhibition enzyme-linked immunosorbent assay; BS, blood smear; CrI, credibility interval; Se, sensitivity; Sp, specificity; P value, Bayesian P value; DIC, Deviance Information Criterion; pD, the effective number of estimated parameters.

The proportion of animals that tested positive for cELISA but were free to the pathogen (challenged-immunized animals) in these endemic areas was about 67% (95% CrI: 62%-73%), which can be interpreted as the proportion of positive-false results coming from cELISA tests in these anaplasmosis endemic areas, meaning that there is a high proportion of immunised animals.

## Discussion

In Ecuador, more than 75% of cattle herds are situated in areas either infested or suitable to the development of cattle ticks [66]. Consequently, TBDs and anaplasmosis in particular, are commonly reported among cattle farmers; yet diagnosing them accurately without laboratory confirmation remains challenging. Despite numerous studies on the prevalence of anaplasmosis in the country, published data on the sensitivity and specificity of serological tests and the true prevalence of the disease still need to be concluded. This highlights the significance of our research, which represents the first reported study employing a Bayesian analysis framework to estimate the true prevalence, sensitivity, and specificity of mPCR, cELISA, and BS tests in an endemic area.

Anaplasmosis presents several diagnostic challenges, including non-specific clinical signs, multiple disease presentations (mild, acute, peracute, and chronic), presence of co-infections, and limitations of serological tests [15,19,20,67,68]. Sub-inoculation of *Anaplasma*-infected red blood cells in splenectomized calves has been considered the “gold standard” for determining persistent *A. marginale* infections in cattle but it is not a routine test and is impractical on a large scale evaluation [67,69].

Bovine anaplasmosis is conventionally diagnosed by the identification of *Anaplasma* spp. in Giemsa-stained BS from clinically suspect animals during the acute phase of the disease. However, this method has several disadvantages, such as requiring experienced personnel and being tedious and time-consuming. In addition, it is not useful for detecting carriers (challenged-immunized animals) and presymptomatic animals; and does not distinguish between *A. marginale* and *A. centrale* [70,71]. In this study the BS method shows a reduced specificity of only 20%, attributable to a high proportion of false positive animals. This limitation is attributed to the operator skills when performing the technique (good smear preparation, proper staining, and a well-trained microscopist), which may result in the unintended misidentification of inclusion bodies unrelated to *A. marginale* [70,72].

Molecular methods have been developed with high sensitivity and specificity; the polymerase chain reaction (PCR) assay is commonly used to identify and differentiate the *Anaplasma* species. PCR additions allow early detection during the prepatent period and identification of an “active” infection at a single time-point [2]. The high cost of reagents and equipment, the need for skilled personnel, and the time involved in performing these tests are not practical for large-scale surveillance [67,70]. A cost-effective alternative is the use of pools: if a pool is positive, one goes back to the original constituent samples to directly count the number of positives [26,29,73]. The model employed in this study incorporated this aspect, obtaining a high sensitivity (98%) and specificity (100%) for the mPCR technique. These results are in line which previous studies employing individual analysis [16,50,74], indicating the feasibility of this alternative.

Currently, the cELISA test is based on the detection of antibodies specifically directed towards major surface protein 5 (MSP-5), and it is one of the most widely used diagnostic techniques used as a screening test to detect cattle infected with *A. marginale* due to its practicality and cost-effectiveness [67]. Despite its established high sensitivity (96%) and specificity (95%) in non-endemic environments [67,75,76], our study, that was conducted in endemic areas uncovered a significant discrepancy. While sensitivity remained robust at 99% (95% CrI: 97-100%), specificity drastically declined to 2% (95% CrI: 1-4%). While a positive ELISA result confirms the presence of the pathogen at some time, as it detects antibodies, it does not necessarily mean that the pathogen is present by the time the test is performed [2,77]. Molloy et al. (1999) [78], using a competitive inhibition ELISA, reported that this test did not clearly differentiate between infected and non-infected populations in herds in *A. marginale* endemic areas. In the present study, the Bayesian model also estimated the proportion of ELISA-positive animals, even if they were not currently infected. The proportion of carrier, persistently infected cattle, or challenge-immunized animals, as they were called in this study, was approximately 70% (67% [95% CrI: 62%-73%]). Thus, in this study, we attribute the lower specificity of the ELISA in this study to the abundant presence of challenge-immunized animals, which possess lifelong immunity and remain asymptomatic following the challenge exposure.

In endemic areas, the transmission of *A. marginale* may occur via the transplacental route, and carrier cows may transfer antibodies through colostrum, providing the calf with passive protection for at least three months [8,9,79]. In these areas, calves are exposed to a continuous challenge of infected ticks throughout the year. They contract natural infections and often show mild symptoms or remain asymptomatic, developing immunity that could last a lifetime [80–82]. A study carried out in endemic areas of Ecuador determined that *A. marginale* prevalence in *R. microplus* was 27% [11]. Persistently infected cattle are not robustly immune to reinfection but robustly protected from experiencing clinical signs, and serve as major reservoirs of infection for mechanical, biological, or vertical transmission, contributing significantly to maintaining the state of endemicity [4,5,67,71,75,76,83]. Therefore, confirming a positive cELISA result with PCR in the absence of clinical disease or a history of endemic anaplasmosis is advisable [67], as a positive ELISA result may be the result of a previous infection and does not necessarily mean the animal is currently infected.

The level of agreement between diagnostic tests was measured through the kappa coefficient, P_pos_, and P_neg_. The kappa coefficient for different test pairs was in slight agreement. Our results suggest a good level of agreement when positive results for cELISA-BS were considered. The agreement was around 50% for sera, which tested positive for cELISA-mPCR and mPCR-BS. Positive animal status may vary depending on whether the form of disease presentation (acute or chronic) [84]. The agreement between different test pairs was low for sera, which tested negative. Consequently, the level of agreement of negative results correlated with the relatively low specificity observed for the mELISA and blood smear, possibly due to the high prevalence of persistently infected cattle or challenged-immunized animals [83]. Animals that tested positive by cELISA but negative by mPCR might be attributed to carrier animals with circulating levels of *A. marginale* below levels detectable by PCR [66]. An *A. marginale* cattle carrier can have bacteremia levels ranging from 0.0025% to 0.000025% of infected erythrocytes. Given that mPCR assays are capable of detecting approximately 0.0001% of infected erythrocytes, mPCR only detects a proportion of carrier animals [85,86]. These results are consistent with a study by Hairgrove et al. (2015) [87] and Gioia et al. (2018) [66], who linked positive cELISA and negative RT-qPCR results to antibodies that remain in the blood after the decrease or clearance of the parasite. In our investigation, no animals tested positive for PCR and negative for cELISA, suggesting the absence of early infections [66,87].

Therefore, the selection of the diagnostic method(s) will depend on what we want to determine. On the one hand, a blood smear can reveal the presence of *Anaplasma* spp. in animals showing acute phase symptoms (clinical cases) to detect bovine anaplasmosis. Similarly, in these cases, the infection can also be diagnosed by serological demonstration of antibodies with confirmation by molecular detection methods [2]. On the other hand, serological tests are particularly useful for epidemiological applications, such as prevalence studies and control programs. Antibody prevalence can indicate the existence of endemic stability, but it is important to interpret individual animal results with caution [78,88,89]. To detect infection in cattle and identify carrier animals for movement to disease-free areas, highly sensitive diagnostic techniques are required [24]. Even end-point PCR may not detect the presence of *Anaplasma* in certain cases, so ELISA using msp5 recombinant protein is a recommended option [23,83]. Our study revealed that mPCR is very sensitive. However, its use alone is not advised, given the possible lack of performance in animals with low intracellular bacteria load. This limitation is not attributed to using pools, as a prior study utilizing the same cELISA commercial kit and real-time PCR revealed that 29% (40/140) of cELISA-positive animals were PCR-positive [90]. Hence, the simultaneous use of ELISA and mPCR is advisable.

The true prevalence of *A. marginale* infection was estimated to be 32% in tropical dairy farms. The estimated true prevalence is lower than the seroprevalence detected by cELISA (98%) and BS (81%) methods but close to mPCR (36%). Preliminary studies on the Ecuadorian coast found similar results (86%) using cELISA [91] and 66% in the Amazonian region using iELISA [92]. Studies conducted in the Ecuadorian Amazon using a nested PCR (nPCR) determined a 44% prevalence for *Anaplasma marginale* [93] and 64% [94]. Although this technique is capable of identifying a greater number of carrier animals, it poses specificity problems for routine use [23]. Other studies using PCR have found a prevalence for *A. marginale* of around 85% on the Ecuadorian coast [77,94,95]. Nevertheless, these studies were confined to a limited number of farms. Moreover, a study conducted by Chávez-Larrea et al. (2023) [96] in two slaughterhouses reported a 60% prevalence of *A. marginale* using PCR. Similarly, the range of apparent prevalence was wide when using blood smears, i.e., from 19 to 39% in the coastal region [97–100] and from 2 to 50% in the Amazon region [93,101–104]. However, the most important finding of this study was the detection of protective antibodies against *Anaplasma marginale* in about 70% of dairy cattle in endemic areas.

The clinical examination determined that most of the animals presented clinical parameters compatible with those of normal adult bovines. Only a small percentage of them (4%) presented body temperatures above 39.5°C. Such elevated body temperature could be attributed to various factors, including potential infectious, inflammatory processes, or stress induced by handling during management [105]. The rare presence of clinical disease outbreaks, added to the antibody prevalence (98%), allows us to presume that the pathogen has reached a state of endemic stability in the study areas [106].

Although the animals in this zone are protected against the disease (challenged-immunized animals), it is common for farmers to implement certain practices to increase productivity on their farms, such as introducing animals from areas where the disease is not present. Kocan et al. (2012) [67] emphasized the importance of maintaining challenge-immunized animals to keep endemic stability and avoid introducing susceptible cattle, which could increase the risk of acute disease. Additionally, since there is no reliable method to eliminate persistent *A. marginale* infections in cattle, animal movement should be restricted as a precautionary measure to protect animals in areas where the disease is not endemic [5,107,108]. Maintaining minimal tick infestations is critical not only for optimizing animal production but also for preserving the endemicity of anaplasmosis, thus playing a paramount role in disease management within these regions [109]. The importance of exposing calves to persistent tick infestation is emphasized [110–112]. This requires housing the calves in paddocks where they can come into contact with ticks, as zero grazing may cause a decline in endemic stability in the area [14,113,114].

## Conclusions

In endemic zones for TBDs, it is imperative to consider the immunological status of the animals under assessment with respect to these TBDs. Diagnostic tests must be validated for application in endemic populations, and it is crucial to interpret test results in conjunction with the exposure history and clinical signs. A significant finding of this study was the estimation that approximately 70% of dairy cattle in those endemic areas harbored protective antibodies against *Anaplasma marginale* in those endemic areas. Such high proportion may stem from persistent exposure with a high pathogen prevalence, as observed in previous studies, or from early primary challenges. The high seroprevalence and infrequent clinical outbreaks suggest that the pathogen has achieved endemic stability. Thus, any control program in these areas must strike the right balance, such as implementing measures for tick control that enhance cattle productivity without compromising the delicate equilibrium required for maintaining endemic stability. In addition, avoiding practices like introducing animals from disease-free areas and/or inadequate calf immunization can disrupt endemic regions, leading to sporadic cases of clinical anaplasmosis. Moreover, it is imperative to highlight that the areas studied are endemic to *A. marginale*, which underlines the potential risk associated with the introduction of other species, such as *A. centrale*, or additional tick-borne pathogens, such as *Babesia* spp. that could cause an outbreak of TBDs. Furthermore, under scenarios of global warming, inadequate knowledge about anaplasmosis management can lead to risky management practices, such as the movement of carrier animals or improper handling of contaminated instruments, which can exacerbate the spread of TBDs to areas where they were previously absent [115].

## Supporting information

S1 Text Competitive, enzyme-linked, immunosorbent assay (cELISA) protocol. (DOCX)

S2 Text Multiplex PCR (mPCR) protocol. (DOCX)

S3 Text Conditional probabilities and test result probabilities, without considering that the mPCR was obtained by pooling. (DOCX)

S4 Text Proportion of challenge-immunized animals (RCIA). (DOCX)

S5 Text WinBUGS code (MODEL 5) used to estimate true prevalence of anaplasmosis and test characteristics for mPCR, cELISA, and blood smear. (DOCX)

S6 Text Kernel density and Gelman Rubin statistic of the parameters estimated with model 5. (DOCX)
