# Supplementary material for: What is the value of testing for tick-borne diseases in cattle in endemic areas? A case study of bovine anaplasmosis
Source: PLoS One. 2025 Mar 12;20(3):e0315202. doi: 10.1371/journal.pone.0315202 (PMC12338951; doi:10.1371/journal.pone.0315202)
Supplement: S1 Text — (DOCX) [file pone.0315202.s001.docx]

**Supporting information 1**

**cELISA Protocol**

1. Thaw the serums to room temperature (23 ± 2°C).
2. Prepare the conjugate: dilute 1 part of the antibody-peroxidase conjugate with 99 parts of the conjugate dilution buffer.
3. Prepare the wash solution: dilute 1 part of the wash solution with 9 parts of distilled water.
4. Load the positive control to the first well and the next 3 wells load with the negative controls.
5. Transfer 50 µl of the controls and serum samples from the transfer microplate to the antigen-coated plate as quickly as possible using a pipette. Tap the microplate so that the samples touch the bottom of the wells.
6. Incubate the microplate for 1 hour at room temperature (23 ± 2°C).
7. Wash: after 1- hour of incubation, remove the contents of the wells and tap the microplate 4 times on absorbent paper. Then wash 2 times with wash solution and tap the microplate on absorbent paper after each wash.
8. Conjugate: add 50µ of the prepared conjugate to each well. Tap the microplate so that the conjugate touches the bottom of the wells.
9. Incubate the microplate for 20 minutes at room temperature (23 ± 2°C).
10. Wash: wash the microplate 4 times.
11. Add 50µl of the substrate solution to each well. Tap the microplate so that the substrate touches the bottom of the wells.
12. Incubate the microplate for 20 minutes at room temperature (23 ± 2°C) and avoid exposure to light.
13. Add 50µl of the wash solution to each well. Tap the microplate so that the substrate mixes with the wash solution at the bottom of the wells.
14. Read the result: read the microplate on the spectrophotometer by setting the device to 620 nm.
