# Supplementary material for: What is the value of testing for tick-borne diseases in cattle in endemic areas? A case study of bovine anaplasmosis
Source: PLoS One. 2025 Mar 12;20(3):e0315202. doi: 10.1371/journal.pone.0315202 (PMC12338951; doi:10.1371/journal.pone.0315202)
Supplement: S2 Text — (DOCX) [file pone.0315202.s002.docx]

**Supporting information 2**

**PCR Protocol**

**Composition of master mix for PCR amplification**

| **Reagent** | **Multiplex PCR** | | | **Individual PCR** | | |
| --- | --- | --- | --- | --- | --- | --- |
|  | **Initial concentration** | **Final concentration** | **Volume per reaction (µL)** | **Initial concentration** | **Final concentration** | **Volume per reaction (µL)** |
| PCR buffer | 5X | 1X | 4 | 5X | 1X | 3 |
| MgCl2 | 25 mM | 1.2 mM | 0.96 | 25 mM | 1.2 mM | 0.72 |
| dNTPs | 25 mM | 0.3 mM | 0.24 | 25 mM | 0.2 mM | 0.12 |
| Ana 19A | 10 µM | 0.2 µM | 0.4 | 10 µM | 0.23 µM | 0.35 |
| Ana 19B | 10 µM | 0.2 µM | 0.4 | 10 µM | 0.23 µM | 0.35 |
| CentF | 10 µM | 0.3 µM | 0.6 | - | - | - |
| CentR | 10 µM | 0.3 µM | 0.6 | - | - | - |
| Taq polymerase | 5 U/µL | 0.14 U/µL | 0.56 | 5 U/µL | 0.033 U/µL | 0.1 |
| DNA | - | - | 5 | - | - | 3 |
| H20 | - | - | 7.24 | - | - | 7.36 |
| **Total volume** |  |  | 20 µL |  |  | 15 µL |

millimolar (mM), micromolar (µM), microliter (µL), units per microliter (U/µL)

**PCR cycling conditions for multiplex PCR and individual PCR of *Anaplasma marginale***

|  | **Multiplex PCR** | | | | **Individual PCR** | | |
| --- | --- | --- | --- | --- | --- | --- | --- |
|  | **Step** | **Temperature** | **Time** | **Number of cycles** | **Temperature** | **Time** | **Number of cycles** |
| **Step 1** | Initial denaturation | 94 | 3 min | 1 | 94 | 4 min | 1 |
| **Step 2** | Denaturation | 94 | 30 s | 35 | 94 | 45 s | 35 |
|  | Annealing | 54 | 45 s |  | 64 | 30 s |  |
|  | Extension | 72 | 1 min |  | 72 | 1 min |  |
| **Step 3** | Final extension | 72 | 10 min | 1 | 72 | 10 min | 1 |

minute, min; second, s
