# Supplementary material for: What is the value of testing for tick-borne diseases in cattle in endemic areas? A case study of bovine anaplasmosis
Source: PLoS One. 2025 Mar 12;20(3):e0315202. doi: 10.1371/journal.pone.0315202 (PMC12338951; doi:10.1371/journal.pone.0315202)
Supplement: S3 Text — (DOCX) [file pone.0315202.s003.docx]

**Supporting information 3**

In the following codes, D+ (D-) indicates that a subject is diseased (disease-free); the actual prevalence of anaplasmosis is represented by TP; the sensitivity and specificity of mPCR by Se1 and Sp1, respectively. Being mPCR (T1), cELISA (T2), blood smear (T3), negative results (0 or -), and positive results (1 or +), the expected cell probabilities (P) under the conditional dependence assumption are:

***Conditional probabilities***

$$\theta_{1}=P\left( D^{+} \right)=TP$$

$$\theta_{2}=P\left( {{T1}^{+}|D}^{+} \right)=Se1$$

$$\theta_{3}=P\left( {{T1}^{-}|D}^{-} \right)=Sp1$$

$$\theta_{4}=P({{T2}^{+}|D}^{+}, {T1}^{+})$$

$$\theta_{5}=P({{T2}^{+}|D}^{+}, {T1}^{-})$$

$$\theta_{6}=P({{T2}^{-}|D}^{-}, {T1}^{-})$$

$$\theta_{7}=P({{T2}^{-}|D}^{-}, {T1}^{+})$$

$$\theta_{8}=P({{T3}^{+}|D}^{+}, {T1}^{+}, {T2}^{+})$$

$$\theta_{9}=P({{T3}^{+}|D}^{+}, {T1}^{+}, {T2}^{-})$$

$$\theta_{10}=P({{T3}^{+}|D}^{+}, {T1}^{-}, {T2}^{+})$$

$$\theta_{11}=P({{T3}^{+}|D}^{+}, {T1}^{-}, {T2}^{-})$$

$$\theta_{12}=P({{T3}^{-}|D}^{-}, {T1}^{-}, {T2}^{-})$$

$$\theta_{13}=P({{T3}^{-}|D}^{-}, {T1}^{-}, {T2}^{+})$$

$$\theta_{14}=P({{T3}^{-}|D}^{-}, {T1}^{+}, {T2}^{-})$$

$$\theta_{15}=P({{T3}^{-}|D}^{-}, {T1}^{+}, {T2}^{+})$$

***Test result probabilities, without considering that the mPCR was obtained by pooling.***

$pr\left[ 1 \right]=P\left( 111 \right)=\theta_{1} \theta_{2} \theta_{4} \theta_{8} +{(1-\theta}_{1}) {(1-\theta}_{3}) {(1-\theta}_{7}) (1-\theta_{15})$

$pr[2]=P(110)=\theta_{1} \theta_{2} \theta_{4} {(1-\theta}_{8})+{(1-\theta}_{1}) {(1-\theta}_{3}) {(1-\theta}_{7}) \theta_{15}$

$pr[3]=P(101)=\theta_{1} \theta_{2} \left( 1-\theta_{4} \right) \theta_{9}+{(1-\theta}_{1}) {(1-\theta}_{3}) \theta_{7} (1-\theta_{14})$

$pr[4]=P(100)=\theta_{1} \theta_{2} \left( 1- \theta_{4} \right) {(1-\theta}_{9})+{(1-\theta}_{1}) {(1-\theta}_{3}) \theta_{7} \theta_{14}$

$pr[5]=P(011)=\theta_{1} {(1-\theta}_{2}) \theta_{5} \theta_{10}+{(1-\theta}_{1}) \theta_{3} {(1-\theta}_{6}) (1-\theta_{13})$

$pr[6]=P(010)=\theta_{1} {(1-\theta}_{2}) \theta_{5} {(1-\theta}_{10}) +{(1-\theta}_{1}) \theta_{3} {(1-\theta}_{6}) \theta_{13}$

$pr[7]=P(001)=\theta_{1} {(1-\theta}_{2}) \left( 1-\theta_{5} \right) \theta_{11}+{(1-\theta}_{1}) \theta_{3} \theta_{6} {(1-\theta}_{12})$

$pr[8]=P(000)=\theta_{1} {(1-\theta}_{2}) (1-\theta_{5}) {(1-\theta}_{11}) + {(1-\theta}_{1}) \theta_{3} \theta_{6} \theta_{12}$
