# Supplementary material for: What is the value of testing for tick-borne diseases in cattle in endemic areas? A case study of bovine anaplasmosis
Source: PLoS One. 2025 Mar 12;20(3):e0315202. doi: 10.1371/journal.pone.0315202 (PMC12338951; doi:10.1371/journal.pone.0315202)
Supplement: S4 Text — (DOCX) [file pone.0315202.s004.docx]

**Supporting information 4**

|  | **D^+^** | | | | **D^-^** | | | |
| --- | --- | --- | --- | --- | --- | --- | --- | --- |
|  | **cELISA^+^** | | **cELISA^-^** | | **cELISA^+^** | | **cELISA^-^** | |
|  | **Blood smear^+^** | **Blood smear ^-^** | **Blood smear^+^** | **Blood smear ^-^** | **Blood smear^+^** | **Blood smear ^-^** | **Blood smear^+^** | **Blood smear ^-^** |
|  | *a* | *b* | *c* | *d* | *e* | *f* | *g* | *h* |
| **mPCR^+^** | P(D^+^∩T1^+^∩ T2^+^∩T3^+^) | P(D^+^∩T1^+^ ∩T2^+^∩ T3^-^) | P(D^+^∩T1^+^∩ T2^-^ ∩T3^+^) | P(D^+^∩T1^+^∩ T2^-^ ∩T3^-^) | P(D^-^∩T1^+^∩ T2^+^ ∩T3^+^) | P(D^-^∩T1^+^ ∩T2^+^∩ T3^-^) | P(D^-^∩T1^+^∩ T2^-^ ∩T3^+^) | P(D^-^∩T1^+^∩ T2^-^ ∩T3^-^) |
|  | *i* | *j* | *k* | *l* | *m* | *n* | *o* | *p* |
| **mPCR^-^** | P(D^+^∩T1^-^ ∩T2^+^ ∩T3^+^) | P(D^+^∩T1^-^ ∩T2^+^ ∩T3^-^) | P(D^+^∩T1^-^ ∩T2^-^ ∩T3^+^) | P(D^+^∩T1^-^ ∩T2^-^ ∩T3^-^) | P(D^-^∩T1^-^ ∩T2^+^ ∩T3^+^) | P(D^-^∩T1^-^ ∩T2^+^ ∩T3^-^) | P(D^-^∩T1^-^ ∩T2^-^ ∩T3^+^) | P(D^-^ ∩T1^-^ ∩T2^-^ ∩T3^-^) |

Legend: D+ (D-), Diseased (Disease-free); Multiplex PCR, mPCR (T1); Competitive-inhibition enzyme-linked immunosorbent assay, cELISA (T2); Blood smear (T3) ; -, Negative results; +, Positive results.

$a=\theta_{1} \theta_{2} \theta_{4} \theta_{8}$

$b=\theta_{1} \theta_{2} \theta_{4} {(1-\theta}_{8})$

$c=\theta_{1} \theta_{2} \left( 1-\theta_{4} \right) \theta_{9}$

$d=\theta_{1} \theta_{2} \left( 1- \theta_{4} \right) {(1-\theta}_{9})$

$e={(1-\theta}_{1}) {(1-\theta}_{3}) {(1-\theta}_{7}) (1-\theta_{15})$

$f={(1-\theta}_{1}) {(1-\theta}_{3}) {(1-\theta}_{7}) \theta_{15}$

$g={(1-\theta}_{1}) {(1-\theta}_{3}) \theta_{7} (1-\theta_{14})$

$h={(1-\theta}_{1}) {(1-\theta}_{3}) \theta_{7} \theta_{14}$

$i=\theta_{1} {(1-\theta}_{2}) \theta_{5} \theta_{10}$

$j=\theta_{1} {(1-\theta}_{2}) \theta_{5} {(1-\theta}_{10})$

$k=\theta_{1} {(1-\theta}_{2}) \left( 1-\theta_{5} \right) \theta_{11}$

$l=\theta_{1} {(1-\theta}_{2}) (1-\theta_{5}) {(1-\theta}_{11})$

$m={(1-\theta}_{1}) \theta_{3} {(1-\theta}_{6}) (1-\theta_{13})$

$n={(1-\theta}_{1}) \theta_{3} {(1-\theta}_{6}) \theta_{13}$

$o={(1-\theta}_{1}) \theta_{3} \theta_{6} {(1-\theta}_{12})$

$p={(1-\theta}_{1}) \theta_{3} \theta_{6} \theta_{12}$

$$Proportion of challenge-immunized animals (RCIA)=\frac{number of false positives}{number of ELISA positives}$$

$$PCIA=\frac{e+f+m+n}{a+b+i+j+e+f+m+n}$$

$$PCIA=\frac{1-\theta_{7}+\theta_{3}\theta_{7}-\theta_{1}+\theta_{1}\theta_{7}-\theta_{1}\theta_{3}\theta_{7}-\theta_{3}\theta_{6}+{\theta_{1}\theta}_{3}\theta_{6}}{\theta_{1}\theta_{2}\theta_{4}+\theta_{1}\theta_{5}-\theta_{1}\theta_{2}\theta_{5}+1-\theta_{7}+\theta_{3}\theta_{7}-\theta_{1}+\theta_{1}\theta_{7}-\theta_{1}\theta_{3}\theta_{7}-\theta_{3}\theta_{6}+{\theta_{1}\theta}_{3}\theta_{6}}$$
