# Supplementary material for: What is the value of testing for tick-borne diseases in cattle in endemic areas? A case study of bovine anaplasmosis
Source: PLoS One. 2025 Mar 12;20(3):e0315202. doi: 10.1371/journal.pone.0315202 (PMC12338951; doi:10.1371/journal.pone.0315202)
Supplement: S5 Text — (DOCX) [file pone.0315202.s005.docx]

**Supporting information 5**

**Appendix 5.1 presents the** **WinBUGS code (MODEL 5) used to estimate true prevalence of anaplasmosis and test characteristics for mPCR, cELISA, and blood smear.**

anaplasma_animal <- model {

r[1:8] ~ dmulti(pr[1:8], n)

th1prime <- th[1]/(1-pow(1-th[1], k))

for (i in 1:k)

{

se_p[i] <- (1 - pow(1 - th[2], i)) * exp(logfact(k))/(exp(logfact(i))*exp(logfact(k-i))) * pow(th[1], i) * pow(1-th[1], k-i)

}

ap <- th1prime * sum(se_p[]) / (1 - pow(1-th[1], k))

pr[1] <- ap * th[2] * th[4] * th[8]

pr[2] <- ap * th[2] * th[4] * (1-th[8])

pr[3] <- ap * th[2] * (1-th[4]) * th[9]

pr[4] <- ap * th[2] * (1-th[4]) * (1-th[9])

pr[5] <- ap * (1-th[2]) * th[5] * th[10] + (1-ap) * (1-th[6]) * (1-th[13])

pr[6] <- ap * (1-th[2]) * th[5] * (1-th[10]) + (1-ap) * (1-th[6]) * th[13]

pr[7] <- ap * (1-th[2]) * (1-th[5]) * th[11]

pr[8] <- ap * (1-th[2]) * (1-th[5]) * (1-th[11]) + (1-ap) * th[6]

r2[1:8] ~ dmulti(pr[1:8],n)

for (i in 1:8)

{

d[i] <- r[i]*log(max(r[i],1)/(pr[i]*n))

d2[i] <- r2[i]*log(max(r2[i],1)/(pr[i]*n))

}

bayesp <- step(sum(d[]) - sum(d2[]))

tp <- th[1]

se[1] <- th[2]

sp[1] <- th[3]

se[2] <- th[2] * th[4] + (1-th[2]) * th[5]

sp[2] <- th[6]

se[3] <- th[2] * (th[4] * th[8] + (1-th[4]) * th[9]) + (1-th[2]) * (th[5] * th[10] + (1-th[5]) * th[11])

sp[3] <- th[6] + (1-th[6]) * th[13]

RCIA<- (1 - th[7] + (th[3]*th[7]) - th[1] + (th[1]*th[7]) + (th[1]*th[3]) - (th[1]*th[3]*th[7]) - (th[3]*th[6]) - (th[1]*th[3]) + (th[1]*th[3]*th[6]) ) / ( (th[1]*th[2]*th[4]) + (th[1]*th[5]) - (th[1]*th[2]*th[5]) + 1 - th[7] + (th[3]*th[7]) - th[1] + (th[1]*th[7]) + (th[1]*th[3]) - (th[1]*th[3]*th[7]) - (th[3]*th[6]) - (th[1]*th[3]) + (th[1]*th[3]*th[6]) )

th[1] ~ dunif(0.00, 1.00)

th[2] ~ dunif(0.95, 1.00)

th[3]<-1

th[4] ~ dunif(0.00, 1.00)

th[5] ~ dunif(0.00, 1.00)

th[6] ~ dunif(0.00, 1.00)

th[7] ~ dunif(0.00, 1.00)

th[8] ~ dunif(0.00, 1.00)

th[9] ~ dunif(0.00, 1.00)

th[10] ~ dunif(0.00, 1.00)

th[11] ~ dunif(0.00, 1.00)

th[12]<-1

th[13] ~ dunif(0.00, 1.00)

th[14] ~ dunif(0.00, 1.00)

th[15] ~ dunif(0.00, 1.00)

}

list(r=c(192,44,0,0,325,70,0,10),n=641, k=5)

**Appendix 5.2 presents the unrestricted WinBUGS code used to estimate true prevalence of anaplasmosis and test characteristics for mPCR, cELISA, and blood smear.**

anaplasma <- model {

r[1:8] ~ dmulti(pr[1:8], n)

th1prime <- th[1]/(1-pow(1-th[1], k))

for (i in 1:k)

{

se_p[i] <- (1 - pow(1 - th[2], i)) * exp(logfact(k))/(exp(logfact(i))*exp(logfact(k-i))) * pow(th[1], i) * pow(1-th[1], k-i)

}

se_p_tot <- sum(se_p[]) / (1 - pow(1-th[1], k))

ap <- th[1] * se_p_tot / (1-pow(1 - th[1] * se_p_tot, k))

pr[1] <- ap * th[2] * th[4] * th[8] + (1-ap) * (1-th[3]) * (1-th[7]) * (1-th[15])

pr[2] <- ap * th[2] * th[4] * (1-th[8]) + (1-ap) * (1-th[3]) * (1-th[7]) * th[15]

pr[3] <- ap * th[2] * (1-th[4]) * th[9] + (1-ap) * (1-th[3]) * th[7] * (1-th[14])

pr[4] <- ap * th[2] * (1-th[4]) * (1-th[9]) + (1-ap) * (1-th[3]) * th[7] * th[14]

pr[5] <- ap * (1-th[2]) * th[5] * th[10] + (1-ap) * th[3] * (1-th[6]) * (1-th[13])

pr[6] <- ap * (1-th[2]) * th[5] * (1-th[10]) + (1-ap) * th[3] * (1-th[6]) * th[13]

pr[7] <- ap * (1-th[2]) * (1-th[5]) * th[11] + (1-ap) * th[3] * th[6] * (1-th[12])

pr[8] <- ap * (1-th[2]) * (1-th[5]) * (1-th[11]) + (1-ap) * th[3] * th[6] * th[12]

r2[1:8] ~ dmulti(pr[1:8],n)

for (i in 1:8)

{

d[i] <- r[i]*log(max(r[i],1)/(pr[i]*n))

d2[i] <- r2[i]*log(max(r2[i],1)/(pr[i]*n))

}

bayesp <- step(sum(d[]) - sum(d2[]))

tp <- th[1]

se[1] <- th[2]

sp[1] <- th[3]

se[2] <- th[2] * th[4] + (1-th[2]) * th[5]

sp[2] <- th[3] * th[6] + (1-th[3]) * th[7]

se[3] <- th[2] * (th[4] * th[8] + (1-th[4]) * th[9]) + (1-th[2]) * (th[5] * th[10] + (1-th[5]) * th[11])

sp[3] <- th[3] * (th[6] * th[12] + (1-th[6]) * th[13]) + (1-th[3]) * (th[7] * th[14] + (1-th[7]) * th[15])

RCIA<- (1 - th[7] + (th[3]*th[7]) - th[1] + (th[1]*th[7]) + (th[1]*th[3]) - (th[1]*th[3]*th[7]) - (th[3]*th[6]) - (th[1]*th[3]) + (th[1]*th[3]*th[6]) ) / ( (th[1]*th[2]*th[4]) + (th[1]*th[5]) - (th[1]*th[2]*th[5]) + 1 - th[7] + (th[3]*th[7]) - th[1] + (th[1]*th[7]) + (th[1]*th[3]) - (th[1]*th[3]*th[7]) - (th[3]*th[6]) - (th[1]*th[3]) + (th[1]*th[3]*th[6]) )

th[1] ~ dunif(0,1)

th[2] ~ dunif(0,1)

th[3] ~ dunif(0,1)

th[4] ~ dunif(0,1)

th[5] ~ dunif(0,1)

th[6] ~ dunif(0,1)

th[7] ~ dunif(0,1)

th[8] ~ dunif(0,1)

th[9] ~ dunif(0,1)

th[10] ~ dunif(0,1)

th[11] ~ dunif(0,1)

th[12] ~ dunif(0,1)

th[13] ~ dunif(0,1)

th[14] ~ dunif(0,1)

th[15] ~ dunif(0,1)

}

list(r=c(185,41,0,0,315,69,0,10),n=620, k=5)

|  | **D^+^** | | | | **D^-^** | | | |
| --- | --- | --- | --- | --- | --- | --- | --- | --- |
|  | **cELISA^+^** | | **cELISA^-^** | | **cELISA^+^** | | **cELISA^-^** | |
|  | **Blood smear^+^** | **Blood smear ^-^** | **Blood smear^+^** | **Blood smear ^-^** | **Blood smear^+^** | **Blood smear ^-^** | **Blood smear^+^** | **Blood smear ^-^** |
|  | *a* | *b* | *c* | *d* | *e* | *f* | *g* | *h* |
| **mPCR^+^** | P(D^+^∩T1^+^∩ T2^+^∩T3^+^) | P(D^+^∩T1^+^ ∩T2^+^∩ T3^-^) | P(D^+^∩T1^+^∩ T2^-^ ∩T3^+^) | P(D^+^∩T1^+^∩ T2^-^ ∩T3^-^) | P(D^-^∩T1^+^∩ T2^+^ ∩T3^+^) | P(D^-^∩T1^+^ ∩T2^+^∩ T3^-^) | P(D^-^∩T1^+^∩ T2^-^ ∩T3^+^) | P(D^-^∩T1^+^∩ T2^-^ ∩T3^-^) |
|  | *i* | *j* | *k* | *l* | *m* | *n* | *o* | *p* |
| **mPCR^-^** | P(D^+^∩T1^-^ ∩T2^+^ ∩T3^+^) | P(D^+^∩T1^-^ ∩T2^+^ ∩T3^-^) | P(D^+^∩T1^-^ ∩T2^-^ ∩T3^+^) | P(D^+^∩T1^-^ ∩T2^-^ ∩T3^-^) | P(D^-^∩T1^-^ ∩T2^+^ ∩T3^+^) | P(D^-^∩T1^-^ ∩T2^+^ ∩T3^-^) | P(D^-^∩T1^-^ ∩T2^-^ ∩T3^+^) | P(D^-^ ∩T1^-^ ∩T2^-^ ∩T3^-^) |

$$Tasa de individuos naturalmente protegidos=\frac{number of false positives}{numero de positivos a ELISA}$$

$$Tasa de falsos positivos=\frac{e+f+m+n}{a+b+i+j+e+f+m+n}$$

$pr\left[ 1 \right]=P\left( 111 \right)=\theta_{1} \theta_{2} \theta_{4} \theta_{8} +{(1-\theta}_{1}) {(1-\theta}_{3}) {(1-\theta}_{7}) (1-\theta_{15})$

$pr[2]=P(110)=\theta_{1} \theta_{2} \theta_{4} {(1-\theta}_{8})+{(1-\theta}_{1}) {(1-\theta}_{3}) {(1-\theta}_{7}) \theta_{15}$

$pr[3]=P(101)=\theta_{1} \theta_{2} \left( 1-\theta_{4} \right) \theta_{9}+{(1-\theta}_{1}) {(1-\theta}_{3}) \theta_{7} (1-\theta_{14})$

$pr[4]=P(100)=\theta_{1} \theta_{2} \left( 1- \theta_{4} \right) {(1-\theta}_{9})+{(1-\theta}_{1}) {(1-\theta}_{3}) \theta_{7} \theta_{14}$

$pr[5]=P(011)=\theta_{1} {(1-\theta}_{2}) \theta_{5} \theta_{10}+{(1-\theta}_{1}) \theta_{3} {(1-\theta}_{6}) (1-\theta_{13})$

$pr[6]=P(010)=\theta_{1} {(1-\theta}_{2}) \theta_{5} {(1-\theta}_{10}) +{(1-\theta}_{1}) \theta_{3} {(1-\theta}_{6}) \theta_{13}$

$pr[7]=P(001)=\theta_{1} {(1-\theta}_{2}) \left( 1-\theta_{5} \right) \theta_{11}+{(1-\theta}_{1}) \theta_{3} \theta_{6} {(1-\theta}_{12})$

$pr[8]=P(000)=\theta_{1} {(1-\theta}_{2}) (1-\theta_{5}) {(1-\theta}_{11}) + {(1-\theta}_{1}) \theta_{3} \theta_{6} \theta_{12}$
