# Supplementary material for: What is the value of testing for tick-borne diseases in cattle in endemic areas? A case study of bovine anaplasmosis
Source: PLoS One. 2025 Mar 12;20(3):e0315202. doi: 10.1371/journal.pone.0315202 (PMC12338951; doi:10.1371/journal.pone.0315202)
Supplement: S6 Text — (DOCX) [file pone.0315202.s006.docx]

**Supporting information 6.**

**Kernel density and Gelman Rubin statistic of the parameters estimated with model 5**

In the following graphs generated in WinBUGS, the sensitivity and specificity of mPCR are represented by y se[1] and sp[1], respectively, of cELISA by se[2] and sp[2] respectively, and of blood smear by se[3] and sp[3] respectively. The true prevalence of anaplasmosis is represented by th[1], and the rate of challenge-immunized animals by RCIA.

**MODEL 5**

***Kernel density***

***Gelman Rubin statistic***
